# Parental information-seeking behaviour in childhood vaccinations

**DOI:** 10.1186/1471-2458-13-1219

**Published:** 2013-12-21

**Authors:** Irene A Harmsen, Gemma G Doorman, Liesbeth Mollema, Robert AC Ruiter, Gerjo Kok, Hester E de Melker

**Affiliations:** 1 National Institute for Public Health and the Environment (RIVM), Centre for Infectious Disease Control, P.O. Box 1 3720, BA, Bilthoven, the Netherlands; 2 Department of Work & Social Psychology, Maastricht University, Maastricht, the Netherlands; 3 Department of Earth and Life Sciences, VU University of Amsterdam, Amsterdam, the Netherlands

**Keywords:** Information seeking, Information need, Internet, Reasoned action approach, Health communication, Vaccination

## Abstract

**Background:**

People want to be well informed and ask for more information regarding their health. The public can use different sources (i.e. the Internet, health care providers, friends, family, television, radio, and newspapers) to access information about their health. Insight into the types and sources of vaccine related information that parents use, and reasons why they seek extra information is needed to improve the existing information supply about childhood vaccinations.

**Methods:**

Dutch parents with one or more children aged 0–4 years received an online questionnaire (N = 4000) measuring psychosocial determinants of information-seeking behaviour and self-reports of types and sources of vaccine information searched for (response rate 14.8%). We also tested two invitation approaches (i.e., reply card versus Internet link in invitation letter) to observe the difference in response rate.

**Results:**

Almost half of the parents (45.8%) searched for extra information. Of all the respondents, 13% indicated they had missed some information, particularly about side effects of vaccines (25%). Intention to search for vaccination information was influenced by positive attitude and perceived social norm towards information-seeking behaviour. There was no difference in the response rate between the two invitation approaches.

**Conclusions:**

The information provided by the National Immunization Programme (NIP) might be sufficient for most parents. However, some parents mentioned that they did not receive enough information about side effects of vaccinations, which was also the topic most searched for by parents. Public Health Institutes (PHIs) and child healthcare workers should therefore be aware of the importance to mention this aspect in their communication (materials) towards parents. The PHIs must ensure that their website is easy to find with different search strategies. Since the child healthcare worker is perceived as the most reliable information source, they should be aware of their role in educating parents about the NIP.

## Background

Nowadays, individuals take an active role in managing their own health. People want to be well informed and ask for more information regarding their health [1]. The public can use different sources (i.e. the Internet, health care providers, friends, family, television, radio and newspapers) to access information about their health [2-7]. Although physicians remain the most highly trusted information sources [8], the use, popularity and perceived importance of the Internet to get health information is rising [9]. Advantages of the use of Internet to search for information are the widespread access, anonymity, social support, and the ability to tailor information to one’s needs [10]. Another advantage might be the large amount of available information. However, this also might be a disadvantage because of the difficulty to understand all the provided information and to know what information is objective and trustworthy [11,12].

Childhood vaccination is a health topic that is highly searched [13,14]. Most parents (79.6%) use 2 to 6 sources when searching for information about childhood vaccination [15]. Different studies have shown that healthcare providers are perceived as the most common and trusted sources of vaccine related information [16,17]. Jones et al. (2012) [15] and Downs et al. (2008) [12] showed that when parents did not view their child healthcare provider as a reliable source of information, they were more likely to obtain information about vaccination through the Internet with the help of search engines [18]. The use of Internet to search for vaccine related information was clearly visible during the pandemic influenza outbreak in 2009 in the Netherlands, when 56% of the acceptors of H1N1 influenza vaccine and 75% of decliners of the H1N1 vaccine searched for information on the Internet [19]. Bults et al. [19] showed that 22% of the vaccine acceptors and 25% of the vaccine decliners visited Internet sites that were critical of vaccination. Anti-vaccination messages are more widespread on the Internet than in other media [20]. These anti-vaccination messages might negatively influence parents’ vaccination decisions [21,22]. Lehman et al. (2013) [23] showed that social media more critically evaluated (influenza) vaccination information than news media.

Some parents feel under informed and report a lack of vaccine-related knowledge [24,25]. Hak et al. (2005) [26] showed that among Dutch parents, 89% want an improvement of the current vaccination education, and that they would like to be educated by health care workers. In the Netherlands, parents receive oral information about the National Immunization Programme (NIP) when a nurse of the child welfare centre visits the parents at their home when their infant is about two weeks old. When the child is 4–6 weeks old, parents receive through surface mail a brochure with information about vaccines, diseases, the vaccination schedule, side effects, and with a reference to the website of the National Institute of Public Health and the Environment (RIVM). When children are 8 weeks old, they will get their first vaccination at the child welfare centre, where they also receive health check-ups. In the Netherlands, the NIP is managed by the RIVM, is voluntary and free of charge, with an overall coverage of 95% in 2012 [27].

So far, no systematic research has been done in the Netherlands about parental information-seeking behaviour in childhood vaccinations. We therefore conducted a quantitative study to get more insight in parents’ information seeking behaviour about the NIP and in their information need. Insight into topics and sources of vaccine related information that parents use, and reasons why they seek extra information is needed to improve existing information supply about the NIP. In particular, we would like to get more insight in the psychosocial determinants that influence the intention of parents to search for information about the NIP. Insight in these determinants might be useful to explore the drivers of parents to search for information.

We also tested in this study two invitation approaches to observe the difference in response rate. Research showed that different invitation approaches resulted in different response rates. A systematic review of Edwards et al. (2002) [28] showed that when surveys were sent by recorded delivery, when stamped return envelopes were used, participants were contacted before sending questionnaires, and when a reminder was used, response was more likely. Since we had used an online questionnaire, we were interested in what kind of invitation method was most effective: sending an invitation letter with a reply card where parents could fill in their e-mail address and then received an e-mail with an Internet link to access the questionnaire, or an invitation letter with an Internet link to the questionnaire.

### Theoretical framework

To study the psychosocial determinants that influence parents’ intention to seek information about childhood vaccination, we used the Reasoned Action Approach (RAA) of Fishbein and Ajzen (2010) [29], which is the most recent formulation of the Theory of Planned Behaviour (TPB) [30]. The TPB is a well-founded theory and often used in health behaviour research [31-33] and in information seeking behaviour studies [34-37].

The RAA indicates that intention, the desire to perform certain behaviour, is determined by attitude towards the behaviour, perceived norm, and perceived behavioural control. Attitude is a positive or negative evaluation of the behaviour. Perceived norm is the perceived social pressure to perform (or not to perform) a behaviour. Perceived behavioural control is an assessment of the individual’s confidence in being able to perform the behaviour [29]. The direct measures attitude, perceived norm and perceived control are determined in turn by behavioural, normative and control beliefs. These beliefs constitute specific expectations about the outcomes of the behaviour, anticipated support from significant others, and barriers and facilitators to perform the behaviour.

Behavioural beliefs (i.e., behavioural belief strength and outcome evaluation) are the indirect measures of attitude and are beliefs of an individual about the outcome of performing a particular behaviour. Normative beliefs (i.e. injunctive normative beliefs and descriptive normative beliefs) are the indirect measures of perceived norm. The injunctive normative beliefs include injunctive normative belief strength and motivation to comply, which refers to beliefs that particular referents approve or not approve behaviour. The descriptive normative beliefs include the descriptive normative belief strength and the identification with the referent. These descriptive normative beliefs refer to a belief of an individual that a particular referent will perform or not perform the behaviour. Control beliefs (i.e. the power of control and control belief strength) are indirect measures for perceived control. These beliefs refer to particular factors that could facilitate or impede performance of the behaviour [29].

## Methods

### Design & procedure

This cross-sectional study design by means of an online self-reported questionnaire were offered to a sample of 4000 parents who lived in the Netherlands, with at least one child aged 0–4 years, randomly selected from Praeventis, the national database for vaccination registration [38].

Two invitation approaches were used and parents were randomly assigned to one of those two. In one approach, parents received an invitation letter regarding the study objectives and procedures, and could inform the researchers whether they wished to participate by sending a reply-card with their e-mail address. Parents who did so then received an e-mail with an Internet link to access the questionnaire. The other approach consisted of the same invitation letter, with an Internet link. Parents could type the Internet link in their web browser to get direct access to the questionnaire. The aim of these two invitation approaches was to evaluate which resulted in the highest response rate. After three weeks, reminder letters with the Internet link to the questionnaire were sent to all of the 4000 parents. Participants were assured of their privacy and confidentiality of their responses in the invitation letter and at the start of the questionnaire. Those who participated gave their informed consent by filling out the questionnaire. This study was approved by the Maastricht University’s Ethics Research Board.

### Questionnaire

The demographic variables gender, age, country of birth, education, household income, number of children in the family and self-reported vaccination status of the children were measured with appropriate items. The other items were based on 7-point Likert scales with the end-points labelled as 1 = totally disagree and 7 = totally agree. Items were averaged into one single concept when they showed sufficient internal consistency (Cronbach’s alpha *α* > .60 or Pearson correlation coefficient *r >* .50 with two items). Evaluation of the personal experience with the NIP was measured with one item (i.e., ‘How is your general experience with the NIP?’ ‘Very bad-very good’). Two items measured consideration: whether immunization of the child is perceived as self-evident, and the amount of thought given to the process of vaccination decision making (i.e., ‘Vaccinating my child is self-evident’ and ‘I don’t have to think long about vaccinating my child.’ , ‘Totally disagree - totally agree’; r = .75). Attitude towards the NIP was measured with three semantic differentials (i.e. ‘I think the NIP is … very bad - very good’; ‘very unimportant - very important’; ‘very unreliable - very reliable’; α = .88).

To get insight in the information need of parents about the NIP, parents were asked multiple-choice questions at what time, from whom and how they would like to receive information and if they missed any information about the NIP. Parents were also asked if they have searched for information about the NIP in the past (yes-no question). Only parents who indicated they had already searched for information got access to the last part of the questionnaire. This part consisted of multiple choice questions about where and when parents had searched for information, which information sources they had used, and whether these information sources had influenced their choice to vaccinate and their opinion about the NIP (self-reported).

The psychosocial determinants of information seeking were based on 7-point Likert scales with the end-points labelled as 1 = totally disagree and 7 = totally agree. Intention was measured with three items (i.e., ‘I expect that I will search for information about the NIP in the future’ , ‘I intend to search for information about the NIP in the future’ , ‘It is likely that I will search for information about the NIP in the future’; *α* = .94). Attitude was measured by three semantic differentials (i.e., ‘Information seeking about the NIP, I think is…very bad-very good’; ‘very unwise-very wise’; ‘very unimportant-very important’; *α* = .83). Perceived norm was measured with three items (i.e., ‘Most people around me think I should search for information about the NIP’; ‘Most people who are important to me think I should search for information about the NIP’ , ‘Most people similar to me think I should search information about the NIP’; *α* = .86). Perceived control was measured by three items (i.e., ‘I can judge what reliable information about vaccinations is’ , ‘After reading all the pros and cons about vaccinations, I can choose whether or not I will vaccinate my child’ , ‘I can find the information that I am searching for’; *α* = .85). Table 1 shows the items of the indirect measures of the RAA.

**Table 1:** Difference between the beliefs of high and low intenders beliefs, based on a median split of intention

**Indirect measures**	**Items**	**Low Intenders (n = 338) M (SD)**	**High Intenders (n = 254) M (SD)**	**p-value**
Behavioural belief strength	- Finding information about the NIP increases my knowledge	4.84 (1.53)	4.97 (1.05)	.35
	- By searching for information about the NIP, it will be more easy for me to make a choice whether to vaccinate my child or not	3.99 (1.69)	4.49 (1.31)	<.01
	- By searching for information about the NIP, I will be more easy for me to talk with others about vaccination	4.25 (1.70)	4.51 (1.09)	.11
Outcome evaluation	- Making a choice about whether or not to vaccinate my child, I think is..*Very bad – Very good*	4.85 (1.57)	5.27 (1.39)	<.01
	- Increasing knowledge about the NIP, I think is…*Very bad – Very good*	4.87 (0.95)	5.13 (0.99)	.02
	- Talking about the NIP with others, I think is…*Very bad – Very good*	4.55 (1.06)	4.95 (0.97)	<.001
Injunctive normative belief	- My friends think I should search for information about the NIP	1.71 (0.85)	2.92 (1.13)	<.001
	- My family think I should search for information about the NIP	1.97 (1.12)	2.99 (1.13)	<.001
	- My general practitioner think I should search for information about the NIP	1.87 (1.06)	2.96 (1.12)	<.001
	- My nurse/doctor at the child welfare centre think I should search for information about the NIP	2.05 (1.26)	3.14 (1.25)	<.001
	- Other parents in my environment with young children (0–4 years) think I should search for information about the NIP.	1.90 (1.01)	3.01 (1.16)	<.001
Descriptive normative belief strength	- Most of my friends search for information about the NIP	2.15 (1.22)	3.16 (1.09)	<.001
	- Other parents in my environment with young children (0–4 years) search for information about the NIP	3.05 (1.36)	3.64 (1.04)	<.001
Power of control	- If I want, I know for sure that I am able to find information about the NIP	5.90 (1.16)	5.69 (1.06)	.07
	- If I want, I have confident that I am able to find information about the NIP	5.65 (1.23)	5.44 (1.06)	.09
	- If I want, I have enough skills to find information about the NIP	5.78 (1.37)	5.54 (1.15)	.08^.^
Control belief strength	- I can assess what reliable information about vaccines is	4.25 (1.50)	4.33 (1.26)	.61^.^
	- After reading all the pros and cons about vaccines, I can choose whether or not I will vaccinate my child	4.95 (1.60)	4.96 (1.35)	.91
	- I can find the information that I am searching for	5.35 (1.31)	5.26 (1.12)	.49

*Note:* M = Mean, SD = Standard Deviation, scale range: 1–7.

### Data-analysis

To analyse the data, IBM SPSS Statistics version 19.0 was used. Descriptive analysis was used to summarize the demographic characteristics. To find out if gender, age, country of birth, and education were related to intention to seek information, *t*-tests, Pearson correlation coefficients and One-Way ANOVA’s were conducted. The bivariate associations between the psychosocial measures of information seeking behaviour were analysed with Pearson correlation coefficients.

To indicate whether gender, age, country of birth, and education were related with parents’ information need and information seeking behavior, a multivariate logistic regression analysis was performed. Descriptive analysis was used for the information need of parents and for items about the actual information seeking behaviour, and parents’ self-reported influence from the used information sources on their vaccination decision and their opinion of the NIP.

To show the unique contribution of variables to the explanation of intention and the total amount of variance explained in intention, we conducted a linear hierarchical regression analysis. In the first step we included all direct psychosocial measures of the RAA. In the second step we included past behaviour, general attitude about the NIP and consideration, and in the third step we included the demographic variables that showed significant associations with intention (*p <* .01).

To analyse the contribution of the indirect measures in the prediction of the direct measures of the RAA, we conducted separate linear regression analysis for each direct measure. Finally, differences in the beliefs between participants with high and low intentions to search for vaccine related information were analysed. Therefore, the sample was divided into two groups using a median split on the intention measure, and analysis was done with independent-samples t-tests.

## Results

### Response rate

A total of 592 out of 4000 (15%) parents completed the questionnaire. Of the 2000 parents who received the information letter with reply card, 211 (11%) returned the reply card, and 135 of those parents (64%; 6.8% of 2000) completed the questionnaire. Of the other 2000 parents who received an information letter with Internet link, 143 (7.2%) completed the questionnaire. Reminder letters with the Internet-link were sent after three weeks to all the 4000 parents. Before the reminder, 278 (7%) parents completed the questionnaire, and after the reminder, another 314 (8%) parents.

### Descriptive statistics

Of the 592 respondents, 85% were female. The mean age was 35 years (SD = 5.3). 92% were born in the Netherlands, and 54% had finished higher education or university. Half of the respondents (51%) had a household income above modal (modal gross household income per year is €32 500 [39]). Most respondents had two children (47%) while 82% reported that their first child received all the recommended vaccinations within the NIP. 16% were partially vaccinated, and 2% were not vaccinated at all.

Overall, the respondents reported a positive experience with the NIP (M = 5.69, SD = 1.18), a positive general attitude towards the NIP (M = 5.81, SD = 0.89), and that vaccinating their child(ren) was self-evident (M = 5.89, SD = 1.38). A small proportion of the respondents (8.4%) indicated that religious beliefs or another conviction had influenced their opinion about childhood vaccination. Parents who indicated this, stated that religion had the most influence on their opinion (M = 4.74, SD = 2.05).

An independent sample *t*-test found no significant differences between males and females in their intention to search for information (*t* (590) = -1.73, *p* = .47), and no significant difference whether parents were born in the Netherlands or not (*t* (592) = -0.38, *p* = .71). Age (*r* = -.02*, p* = .49) and educational level (*F* (18, 573) = 1.02, *p* = .43) were also not significantly related with intention to search for information.

### Information need of the parents

A logistic regression analysis showed that parents who were born in the Netherlands had a higher need for information than those who were born in another country (OR = 0.42, 95% CI: 0.22 - 0.81). All the other demographic variables (i.e., age (OR = 0.98, 95% CI: 0.94 - 1.03), gender (OR = 1.55, 95% CI: 0.81 – 2.96), education level (OR = 1.14, 95% CI: 0.98 – 1.32)) showed no significant influence to parents’ information need.

Overall, parents indicated that they would like to receive information about the NIP after birth, but before the first vaccination of their child. Most parents (33%) thought the child welfare centre was the most reliable source to provide information about childhood vaccination, followed by the general practitioner (29%), and the RIVM (22%). Parents would like to receive the information in a brochure (63%) or by a letter (55%).

Of all the parents, 13% (n = 79) indicated that they did not receive enough information from the RIVM about the NIP. Of the parents who had missed information, most of them would like to receive more information about possible negative consequences of vaccines (25%) and side effects of vaccines (18%). Other topics parents would like to receive more information about were: the components of the vaccines (10%), reasons for including vaccines in the programme (7%), and counter indications for vaccination when, for example, the child is sick or has a chronic disease (6%).

### Information seeking behaviour

A logistic regression analysis showed that parents who were born in the Netherlands were more likely to search for information than those who were born in another country (OR = 0.31, 95% CI: 0.16 – 0.59). Also higher educated parents (OR = 1.23, 95% CI: 1.10 – 1.38) and parents with an older age (OR = 1.04, 95% CI: 1.00 – 1.08) were more likely to search for information. Gender (OR = 1.38, 95% CI: 0.83 – 2.30) had no significant influence to parents’ information seeking behaviour.

Almost half of the respondents (n = 271, 46%) searched for information about the NIP. More than a third (36%) of the parents started to search for information after the birth of their child and before the first vaccination, followed by 24% of the parents who started to search for information between the vaccination rounds in the NIP. The most common topics that parents searched for were: side effects (82%), vaccines (60%), diseases (56%), and the vaccination schedule (51%). Parents reported that when they found information about the NIP they looked for, they did not search for more NIP related information again (M = 3.75, SD = 1.56).

The information sources that parents used most were: the Internet (41%), the doctor (26%) and nurse (23%) of the child welfare centre, their family (21%) and friends (18%). Among the parents who used the Internet, the website of the RIVM was the most used Internet site (49%), followed by Google and other search engines (29%). Some parents (8%) visited the Internet site of the association of critical vaccination in the Netherlands. Parents who used the website of the RIVM reported that the website had almost no influence on their choice to vaccinate or not (M = 3.17, SD = 1.63), and a small influence on their opinion about the NIP (M = 3.84, SD = 1.56). Parents who consulted the doctor and/or the nurse at the child welfare centre reported that these health care workers had a small influence on their choice to vaccinate or not (doctor: M = 3.52, SD = 1.75; nurse: M = 3.64, SD = 1.73) and on their opinion about the NIP (doctor: M = 3.68, SD = 1.79; nurse: M = 3.90, SD = 1.73). Family and friends were also perceived as not having a big influence on parents’ choice to vaccinate their child or not (family: M = 4.31, SD = 1.76; friends: M = 3.75, SD = 1.64), and on their opinion about the NIP (family: M = 4.19, SD = 1.78; friends: M = 3.69, SD = 1.60). Parents indicated that the information they found was complete (M = 4.91, SD = 1.38) and gave an answer to their questions (M = 4.98, SD = 1.40).

### Direct and indirect measures of intention to search for information

Table 2 shows the means, standard deviations (SD) and Pearson correlations for the direct measures of the RAA. Parents had a positive attitude towards information seeking about the NIP (M = 5.23, SD = 0.90), and perceived a weak pressure from their social environment to search for information (M = 3.18, SD = 1.33). Parents believed they were able to search for information about the NIP (M = 5.72, SD = 1.05).

**Table 2:** Correlation matrix and mean scores (SD) of direct measures of the RAA

	**1.**	**2.**	**3.**	**4.**
1. Intention				
2. Attitude	.44*			
3. Perceived norm	.51*	.38*		
4. Perceived control	.04^n.s.^	.16*	-.04^n.s.^	
Mean score	3.92	5.23	3.18	5.72
SD	1.5	0.8	1.3	1.0

*Note*: * p < .001*,* n.s. = not significant, scale range: 1–7, SD = Standard Deviation.

To get insight in the strength of the association among the direct measures and intention, Pearson correlation were calculated. In terms of effect sizes, small associations are correlations with *r* between .10 and .23; a correlation between .24 and .36 shows a moderate effect, and a correlation of *r* > .37 indicates a large association [40]. The direct measures attitude and perceived norm showed a strong positive association with intention to search for information about the NIP (see Table 2).

Table 3 shows the results of the linear hierarchical regression of intention on the direct measures. The demographic variables gender, age and educational level were not significantly related with intention to search for information, and were therefore not included in the regression analysis. The first column shows the regression with all the direct RAA measures. Attitude and perceived norm are the most powerful significant predictors of intention to search for information about the NIP. The direct measures of the RAA explained 34% of the variance in intention to search for information about the NIP (*R*^2^ =. 34). Adding past behaviour, general attitude about the NIP, and considerations about the choice to vaccinate in the second step showed a negative significant contribution of general attitude about the NIP, and a positive significant contribution of past behaviour (whether parents already searched for information about the NIP) at intention. The amount of thought given to the choice to vaccinate or not (consideration) was not significantly associated to parents’ intention to search for NIP information, while attitude and perceived norm remained significant. By including these measures, the explained variance increased to 36% (*R*^*2*^ = .36).

**Table 3:** Linear hierarchical regression analyses of intention on the direct measures of RAA, past information-seeking behaviour, NIP attitude, and considerations

	***r***	***b***	**SE**	***p-value***	***b***	**SE**	***p-Value***
Attitude information seeking	.44*	0.49	.06	.00	0.42	.07	<.001
Perceived norm	.51*	0.46	.04	.00	0.40	.04	<.001
Perceived control	.04*	0.02	.05	.66	0.05	.05	.27
Past information-seeking behaviour	.29*				0.42	.11	<.001
NIP attitude	-.07^n.s.^				-0.17	.08	.03
Considerations about choice to vaccinate	-.12*				0.02	.05	.62
*R*^2^		.34			.36		<.001
Δ*R*^*2*^					.03		

*Note: b =* unstandardized regression coefficient*, SE* = Standard Error, *R*^*2*^ = explained variance.

*p < 0.001, n.s. = not significant.

Table 4 shows the linear regression analysis of the direct measures on the related indirect measures of the RAA. Finding information to increase knowledge was positively associated with parents’ attitude to search for information. The belief that increasing knowledge about the NIP is good, and that talking about the NIP with others is good, was also positively associated with parents’ attitude. The behavioural beliefs explained 55% (*R*^*2*^ = .55) of the variance in attitude to search for NIP information. The belief that friends, family, the general practitioner, and other parents, think that they should search NIP information was positively associated with perceived norm. Also, the belief that other parents and friends search for NIP information themselves was positively associated with the direct measure perceived norm. The normative beliefs explained 85% (*R*^*2*^ = .85) of the variance in perceived norm to search for NIP information. One item of the control beliefs: ‘If I want, I am confident that I am able to find information about the NIP’ was excluded from the regression analysis because of multi-collinearity. The other control beliefs: having enough skills, and being able to find the wanted information, were positively associated with perceived control. The control beliefs explained 95% (*R*^*2*^ = .95) of the variance in perceived control to search for NIP information.

**Table 4:** Linear regression analyses of direct RAA measures on the related indirect RAA measures

	**Attitude**		
**Behavioural belief strength**	***b***	**S.E.**	***p-value***
Finding information about the NIP increases my knowledge	0.15	.03	<.001
By searching for information about the NIP, it will be more easy for me to make a choice whether to vaccinate my child or not	0.05	.02	.03
By searching for information about the NIP, I will be more easy for me to talk with others about vaccination	0.00	.03	.90
Making a choice about whether or not to vaccinate my child, I think is..*Very bad-Very good*	0.02	.02	.33
Increasing knowledge about the NIP, I think is…*Very bad – Very good*	0.41	.03	<.001
Talking about the NIP with others, I think is…*Very bad – Very good*	0.16	.03	<.001
*R*^2^	.55		
	**Perceived norm**		
**Normative beliefs**	***b***	**S.E.**	***p-Value***
My friends think I should search for information about the NIP	0.08	.03	<.05
My family think I should search for information about the NIP	0.43	.03	<.001
My general practitioner think I should search for information about the NIP	0.08	.03	<.01
My nurse/doctor at the child welfare centre think I should search for information about the NIP	0.00	.02	.99
Other parents in my environment with young children (0–4 years) think I should search for information about the NIP	0.16	.03	<.001
Most of my friends search for information about the NIP	0.09	.02	<.001
Other parents in my environment with young children (0–4 years) search for information about the NIP	0.12	.02	<.001
*R*^2^	.85		
	**Perceived control**		
**Control beliefs**	***b***	**S.E.**	***p*-value**
If I want, I know for sure that I am able to find information about the NIP	0.01	0.02	.47
If I want, I have enough skills to find information about the NIP	0.43	0.01	<.001
I can assess what reliable information about vaccines is	-0.00	0.01	.62
After reading all the pros and cons about vaccines, I can choose whether or not I will vaccinate my child	0.02	0.01	.05
I can find the information that I am searching for	0.10	0.01	<.001
*R*^2^	.95		

*Note: b =* unstandardized regression coefficient*,* SE = Standard Error, *R*^2^ = explained variance.

### Differences in beliefs between high and low intenders

To determine differences in beliefs between those with higher and lower intention to search for vaccine related information, the sample was divided into two groups based on a median split in the intention distribution (median = 4.0), which is presented in Table 1. Parents with a high intention seem to have stronger beliefs that searching for vaccine related information will make it easier to make a decision whether to vaccinate their child or not. Parents with a high intention also felt a higher social pressure of their social environment (friends, family, general practitioner, nurse/doctor child welfare centre) to search for information. Parents with low and high intentions both felt confident to search for information and thought that they were able to find the information they were searching for.

## Discussion

This study shows that most parents believe that health care workers at child welfare centers are the most reliable source to provide information about the NIP, which is in line with previous research [16,41-43]. Parents would like to receive information that is presented in a brochure or folder, which is not in line with a study of Hak et al. (2005) [26], who showed that Dutch parents preferred to be educated by health care workers. Downs et al. (2008) [12] showed that parents often feel under-informed and lack relevant vaccination knowledge, which is not completely in line with this study, where only 13% of the respondents reported not receiving enough information, mainly about side effects and possible negative consequences of childhood vaccination. It seems that the information provided by the RIVM is sufficient for most of the parents.

Although a small amount of the parents indicated to have a need for information, almost half of the respondents (46%) searched for information about the NIP. This is less than the amount of Dutch parents that searched for information during the H1N1 pandemic in 2009 (76% of vaccine decliners and 56% of vaccine acceptors) [19]. The difference might be caused because the H1N1 was a pandemic with a lot of media attention. Parents perceived health care workers as the most reliable source of vaccine related information, but they used the Internet most of the time to search for information, which is also shown in a study of Downs et al. (2008) [12]. This study shows that the information parents searched for most of the time (side effects and possible negative outcomes of vaccination) was the same information they missed from the Public Health Institute (PHI), despite the fact that side effects is a topic that is mentioned in the education material by the PHI in the Netherlands. It might be that the information provided by the PHI about side effects is not enough, or that parents do not read the material well enough and therefore feel under informed. PHIs should therefore try to improve their communication (materials) about childhood vaccination by including more or other information about side effects, and possible negative consequences of childhood vaccination. Healthcare workers should also be aware of the information need of parents, and try to educate them about side effects when parents visit the child healthcare centre.

To get insight into the drivers that influence parents’ intention to search for vaccine related information, we used the Reasoned Action Approach (RAA) of Fishbein and Ajzen (2010) [29]. The measures of the RAA explained 34% of the variance in intention, which is somewhat lower than the average of 41% that was found in a review of the TPB by Godin and Kok (1996) [44]. Although the RAA seems to be a useful theory to explore drivers for vaccine related information seeking behaviour, other factors like: perceived hazard characteristics, affective response to risk, information sufficiency and someone’s personal capacity to learn, which are part of the Risk Information Seeking and Processing (RISP) model of Griffin et al. [45], might increase the explained variance of intention by psychosocial determinants.

Overall, parents had a positive attitude towards information seeking about the NIP and perceived a low influence of their social environment to search for information. Although parents reported a low social pressure to search for information, parents’ social environment seems to have a significant influence on the intention to search for information, which is in line with a study of Clarke et al. (2012) [34]. Searching for information may be driven by people’s social environment, because sharing information may contribute to the feeling of belonging to a group [46].

This study showed that highly educated parents searched for more information and when parents were more negative about the NIP, they were more likely to search for information. Parents indicated that the information they found did not have a big influence at their attitude or intention to vaccinate or not. Although parents indicated that the information they found did not have a big influence, Betsch et al. (2010) [22] showed in an experiment that searching for information might turn out to have a negative influence at parents’ perceptions and intention to vaccinate, especially when they visit vaccine-critical websites. A study in the US [47] showed that when parents search for vaccine related information, out of the 30 sites, 21 were immunisation sites, and 5 of them were classified as anti-vaccination. In addition, Wolfe & Sharp (2005) [48] showed that a search on the word “vaccination” produced more anti-vaccination than pro-vaccination websites. When parents use less specific search terms, they were more likely to find vaccine critical websites [12]. To prevent parents from visiting vaccine-critical websites, Public Health Institutes should be aware of parents information need, try to fulfil this need by improving their communication materials, and assure that their websites are easy to find, even when parents use negative search terms about the NIP. The PHI could also communicate to parents where to find reliable information about childhood vaccination.

Another aim of our study was to investigate two different invitation approaches (i.e., reply card versus Internet link in invitation letter) to test the difference in response rate. The parents who received a reply card had a higher response (11%), but not all parents completed the questionnaire, so at the end, the response rate of the two different approaches was the same (7%). The information letter with the link to the questionnaire is the preferred method, because this is a more time efficient approach compared to reply cards. When time is not an issue, more effort could be done to include all the parents who returned the reply card and did not complete the questionnaire to get a higher response rate. In this study, the response rate was doubled after sending a reminder. The positive effect of a reminder is also shown in other studies [28,49]. We therefore recommend to use a reminder when sending out (online) questionnaires.

This study also has several limitations. First of all, there might be response bias, because the results of this study are based on an overall questionnaire response rate of 15%. Unfortunately no further data was available to compare participating parents with non-participating parents. A second limitation might be that although in the Netherlands 96% of all 12 to 75 year-olds use the internet, [50] only those who had access to the Internet could complete the questionnaire.

## Conclusions

Only 13% of the respondents searched for extra information about the NIP, so the information provided by the NIP might be sufficient for most parents. However, some parents mentioned that they did not receive enough information about side effects of vaccinations. PHIs and child healthcare workers should therefore be aware of the importance to mention this aspect in their communication (materials) towards parents. Because most parents used the search engine on the internet to get vaccine related information, the PHI must ensure that their website is easy to find with different search strategies. This study also showed that parents who were more negative about the NIP were more likely to search for information about the NIP which might result in more negative attitudes because of the chance of visiting anti-vaccination websites. Listening carefully to the needs of parents who are more negative about the NIP and trying to fulfill their information need with reliable sources is therefore important. Because the child healthcare worker is perceived as the most reliable information source, they should be aware of their role in educating parents about the NIP.

## Competing interests

The authors declare that they have no competing interests.

## Authors’ contributions

All authors discussed the study design and research instrument. IH and GD prepared the questionnaire and performed the data collection and data analysis. IH wrote the first version of the manuscript. All authors contributed to the draft of the final manuscript; their remarks were discussed and processed into the final version that was finally approved by all authors. All authors read and approved the final manuscript.

## Pre-publication history

The pre-publication history for this paper can be accessed here:

http://www.biomedcentral.com/1471-2458/13/1219/prepub
